# Does losing reduce the tendency to engage with rivals to reach mates? An experimental test

**DOI:** 10.1093/beheco/arae037

**Published:** 2024-05-03

**Authors:** Chenke Zang, Meng-Han Joseph Chung, Teresa Neeman, Lauren Harrison, Ivan M Vinogradov, Michael D Jennions

**Affiliations:** Division of Ecology and Evolution, Research School of Biology, Australian National University, Canberra, Australian Capital Territory, 2601, Australia; Division of Ecology and Evolution, Research School of Biology, Australian National University, Canberra, Australian Capital Territory, 2601, Australia; Biological Data Science Institute, Australian National University, Canberra Australian Capital Territory, 2601, Australia; School of Biological Sciences, University of East Anglia, Norwich Research Park, Norwich NR4 7TJ, United Kingdom; Division of Ecology and Evolution, Research School of Biology, Australian National University, Canberra, Australian Capital Territory, 2601, Australia; Division of Ecology and Evolution, Research School of Biology, Australian National University, Canberra, Australian Capital Territory, 2601, Australia; Stellenbosch Institute for Advanced Study (STIAS), Wallenberg Centre at Stellenbosch University, Stellenbosch 7600, South Africa

**Keywords:** contests, male–male competition, mosquitofish, sexual selection, winner–loser effect

## Abstract

Male–male contests for access to females or breeding resources are critical in determining male reproductive success. Larger males and those with more effective weaponry are more likely to win fights. However, even after controlling for such predictors of fighting ability, studies have reported a winner–loser effect: previous winners are more likely to win subsequent contests, while losers often suffer repeated defeats. While the effect of winning–losing is well-documented for the outcome of future fights, its effect on other behaviors (e.g. mating) remains poorly investigated. Here, we test whether a winning versus losing experience influenced subsequent behaviors of male mosquitofish (*Gambusia holbrooki*) toward rivals and potential mates. We housed focal males with either a smaller or larger opponent for 24 h to manipulate their fighting experience to become winners or losers, respectively. The focal males then underwent tests that required them to enter and swim through a narrow corridor to reach females, bypassing a cylinder that contained either a larger rival male (competitive scenario), a juvenile or was empty (non-competitive scenarios). The tests were repeated after 1 wk. Winners were more likely to leave the start area and to reach the females, but only when a larger rival was presented, indicating higher levels of risk-taking behavior in aggressive interactions. This winner–loser effect persisted for at least 1 wk. We suggest that male mosquitofish adjust their assessment of their own and/or their rival’s fighting ability following contests in ways whose detection by researchers depends on the social context.

## Teaser

The winner–loser effect occurs when previous winners of fights are more likely to repeat their fighting success. But can the winner–loser effect influence male behaviors in other social contexts? In male mosquitofish, winners increase their aggressiveness in the early stage of male–male competition for mating, and this effect endures for at least 1 wk. Our results indicate a long-lasting influence of a winning/losing experience on behaviors closely related to male mating success.

## Introduction

Males tend to spend more of their lives than do females trying to acquire mates. This difference arises because females usually take longer to breed, making them the rarer sex in the mating pool (Kokko et al. 2012). Consequently, males typically experience intense competition for a limited number of receptive females. This competition drives the sexual selection of traits that elevate a male’s mating and fertilization success (Fromhage and Jennions 2016). Much attention is paid to the evolution of male ornaments that evolve due to female mate choice, but many male traits evolve simply because they are favored when males fight for females (Huntingford and Turner 1987; Vieira and Peixoto 2013), or for resources, like territories (Lee et al. 2022), display sites (Arak 1983), and social status (Hunt et al. 2009) that then increase access to females. Winning fights elevates a male’s reproductive success, which tends to select for larger body size and more effective weaponry (Hellmann et al. 2020). Fights are costly, however, so there is also selection on the ability to assess rivals, judge their relative strength, and only initiate contests that a male has a reasonable chance of winning (Rillich and Stevenson 2019).

Winning males that outcompete rivals often differ from losers in characteristics, such as body size, that affect their resource-holding potential, which is defined by the suite of traits that determine their likelihood of winning contests (Allen and Krofel 2017). In addition, however, the outcome of past fights can affect current contests via a “winner-loser effect” (Hsu and Wolf 1999). Specifically, even after controlling for resource-holding potential, previous victories can increase the likelihood of winning contests, while past defeats can lower the probability of winning subsequent contests (Hsu et al. 2006). This has been attributed to changes in self-assessment of fighting ability (Whitehouse 1997) or phenotypic changes (e.g. in body condition) that are visible to opponents (Obermeier and Schmitz, 2003; Taylor and Jackson, 2003; Rutte et al. 2006). The outcome of male–male contests is therefore not only determined by the difference in inherent resource-holding potential between two males but it is also influenced by information males acquire from previous fights about their own and/or their rival’s net competitive ability (Laland et al. 2011). In addition to directly improving fighting success, such winning/losing experience might affect other behaviors that arise in both non-sexual and sexual social contexts. For example, male *Rivulus* fish that won contests then had increased aggressiveness and were more exploratory (Chang et al. 2012), and losers in crayfish showed a significant decrease in boldness (Mathews 2021). This suggests that winning a contest might improve a male’s resource acquisition, thereby improving his fighting ability.

Detecting a genuine winner–loser effect is challenging because males that win fights often have larger weapons, are bigger-bodied, and have greater access to resources. Variations in inherent resource-holding potential could independently affect both the past and current likelihood of winning a fight (Hsu et al. 2006). Ideally, it is therefore necessary to control for variation in resource-holding potential by experimentally creating winners and losers for males otherwise matched for inherent fighting ability (e.g. using proxies of body or weapon size) and then testing for a difference in their subsequent fighting success (e.g. Harrison et al. 2018). More generally, there is evidence from naturally occurring winners and losers of a pattern of repeated wins and loses consistent with a genuine winner–loser effect in many taxa, ranging from insects to mammals (Hsu et al. 2006; Rutte et al. 2006). This pattern could indicate a causal effect of winning past contests, which increases future fighting success (genuine winner/loser effect), or because some correlates of winning contests are consistent over time (e.g. weapon size).

Given the potential winner–loser effect on broader social interaction, it should be noted, however, that most studies have only detected a winner–loser effect that persists for a short period (Hsu et al. 2006; Dehnen et al. 2022), such as 15–60 min in pumpkinseed sunfish (*Lepomis gibbosus*) (Chase et al. 1994), or 20 min in rusty crayfish (*Orconectes rusticus*) (Bergman et al. 2003). It remains poorly known if winning/losing a contest can have longer-term effects, but there is a small body of evidence for a long-term effect on contest outcome in fish, such as mangrove killifish (*Kryptolebias marmoratus*) (Lan and Hsu 2011). Investigation into the potential for prolonged winner–loser effects on other behaviors in fish, especially those related to mating success is, however, lacking (but see Harrison et al. 2023).

When testing for a winner–loser effect on male mating behavior, accounting for individual variations among focal males is advisable, as different males adopt diverse strategies in conventional contests and mate acquisition. To win fights requires the strategic allocation of resource to weapons or growth (Simmons et al. 2017). Males that experience poor conditions (e.g. low food availability) often invest proportionately less into such traits and are more likely to lose contests. These males benefit little from conventional contests for access to mate, and instead, they often pursue alternative reproductive tactics (Gross 1996; Dougherty et al. 2022). For example, small male Masu salmon (*Oncorhynchus masou*) are more likely than larger males to sneak into spawning nests to release sperm, rather than fight for territories (Watanabe et al. 2008). On the other hand, while males that tend to win contests have more mating opportunities, mating is energetically costly and can even lead to sperm depletion (e.g. Shandilya et al. 2021). Males that are more likely to win fights should, therefore, be strategic when deciding which mating opportunities to pursue. For example, regular winners might be less inclined to expend energy competing for a low-quality female. Given that the benefits of reproductive decisions vary depending on a male’s energy budget, it is important to control for extrinsic factors (e.g. food availability) and intrinsic factors (e.g. body condition) when testing for a winner–loser effect on male mating behavior.

Here, we experimentally tested for a winner–loser effect on how male eastern mosquitofish (*Gambusia holbrooki*) respond to potential rivals to pursue mating opportunities. The eastern mosquitofish, originating from North America, is an invasive species that has successfully colonized waterways worldwide. Female mosquitofish mate multiply and store sperm, and are constantly chased by males who repeatedly try to mate (Pyke 2005; Wilson 2005). Males approach a female from behind, dart forward, and try to insert their gonopodium (i.e. intromittent organ) into her gonoduct (Pilastro et al. 1997). Larger males tend to win male–male contests (Harrison et al. 2023) and chase away rivals (Pilastro et al. 2003) and therefore have greater access to females. Recent experimental studies have documented a winner–loser effect on mating behavior in mosquitofish: previous winners spend more time near females and more often attempt to copulate (Harrison et al. 2018, 2023). We therefore ask whether male mosquitofish can adjust their behavior in response to a history of winning or losing in broader social contexts. Specifically, we test whether previous winners and losers differ in their propensity to engage with a larger rival to reach potential mates, and, if so, whether such an effect can persist for a longer duration (i.e. a week).

To control for potential variations in male body conditions, we experimentally manipulated the fighting history of size-matched focal males. We then compared the behavior of winners and losers given the opportunity to approach prospective mates when they had to swim near a larger rival male, a juvenile fish (control I) or in the absence of a conspecific (control II). We further tested for the persistence of any winner–loser effect by running a second set of trials 1 wk later.

## Methods

### Origin and maintenance of mosquitofish

Juvenile mosquitofish were collected from the wild in Canberra, Australia (35°14ʹ30.1ʹʹS, 149°06ʹ17.0ʹʹE). Juveniles were held in mixed-sex groups in 90 L stock aquaria (~60 fish/tank) until they reached maturity (identified by an elongated anal fin for males and a visible gravid spot for females). Adults were then transferred into single-sex stock aquaria. Fish were kept under a 14:10 L:D photoperiod at 26 ± 1 °C and fed commercial flakes in the morning and *Artemia* nauplii ad libitum in the afternoon for stock tanks, or *Artemia* nauplii ad libitum twice daily for focal fish in individual tanks.

### Experimental design

Adult males were randomly selected from the stock population and anesthetized in ice slurry for 10 s before being photographed. We measured their standard length (SL: the snout tip to the end of the vertebral column) from the photographs using *ImageJ* software (Abramoff et al. 2004). We then manipulated the contest outcome of males by randomly assigning 2 size-matched males to be “winners” or “losers” (< 0.1 mm difference in SL; paired *t*-test, *t* = −0.140, *P* = 0.890; *n* = 46 pairs). To create winners, focal males were housed in individual 4L tanks with a smaller stimulus stock male (paired *t*-test, *t* = 168.25, *P* < 0.001; *n* = 46 difference in SL: 3.37 ± 0.14 mm) for 24 h, while losers were created by housing a focal male with a larger stimulus stock male (paired *t*-test, *t* = −96.635, *P* < 0.001; *n* = 46; difference in SL: 3.09 ± 0.22 mm) for 24 h. Past studies have shown that larger male *G. holbrooki* usually win fights (Harrison et al. 2023). To minimize variation among focal males in their recent contest outcomes in the stock tanks, all focal males were isolated in individual 1 L tanks for at least 2 wk before these experimental pairing. All test tanks were separated by black plastic to prevent visual contact among focal males.

**Fig. 1.**
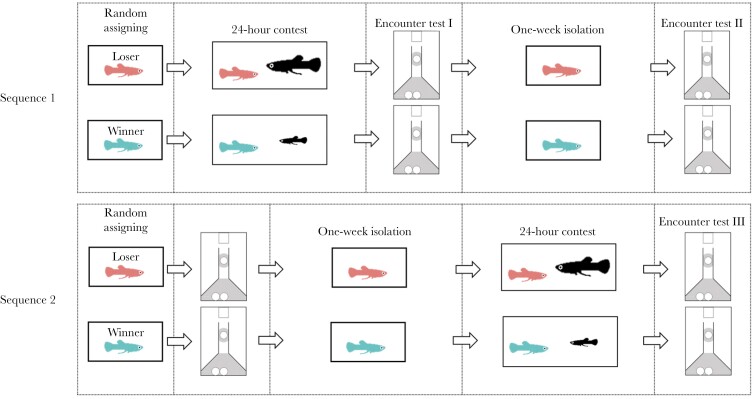
Illustration of the experimental design. A pair of size-matched males (winner and loser) was randomly selected and subjected to the same procedure (sequence 1 or sequence 2). In sequence 1, the males were first exposed to winning or losing contests for 24 h and then immediately underwent their first encounter test (Test I). Subsequently, the males were isolated in individual tanks for a week before their second encounter test (Test II) (*n* = 23 pairs). In sequence 2, males underwent the first encounter test prior to any contest experience. Afterwards these males were transferred into individual tanks for a week before having a winning or losing experience for 24 h. Immediately afterwards they immediately underwent a second encounter test (Test III) (*n* = 23 pairs).

**Fig. 2.**
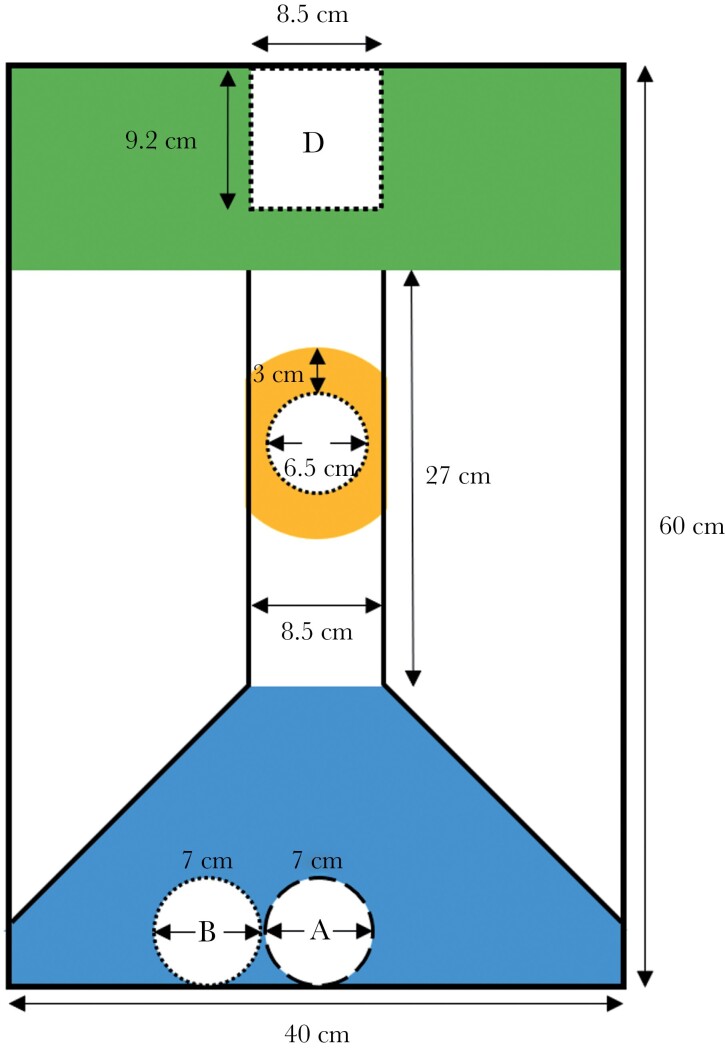
Experimental apparatus for the encounter test. Prior to each trial, a focal male was placed into a mesh cylinder (A), with 2 juveniles were housed in an adjacent transparent cylinder (B). The position of cylinder (B) was rotated between trials (left or right relative to the focal male). Another transparent cylinder (C) in the corridor contained either a large rival male, a juvenile (control), or was empty (control). Four females in a transparent tank (D) were placed at the end of the aquarium in the reward area. The start area (blue), the encounter area (orange: < 3 cm around the transparent cylinder in the corridor), and the reward area (green) are shown.

We used encounter tests (see below) to investigate the effect of winning or losing on how males responded to a larger rival when seeking potential mates. Most studies have typically tested for a short-term effect of winning or losing contests on male behavior (e.g. within a few hours of fighting; Dehnen et al. 2022). Here, we examined whether winners/losers changed their behavior with the time that had elapsed since their previous contest experience. To do this, each male was tested twice in encounter tests: once immediately after the contest period ended (Test I) and then again 1 wk later (Test II). During the 1-wk interval between tests, focal males were socially isolated in their 1 L tank (sequence 1 in Fig. 1; *n* = 23 pairs). To control for any potential effects of time and/or familiarity with the aquarium setup in Test II (Fig. 2), we ran an additional pair of encounter tests with another set of males who did not initially experience staged contests (sequence 2 in Fig. 1; *n* = 23 pairs). After their first encounter test, focal males were isolated in individual 1 L tanks for a week. They were then randomly assigned to become a “winner” or a “loser” for 24 h (as above), after which we recorded these males’ behavior during their second encounter test (Test III). In brief, we combined Tests I and III to test for an initial winner–loser effect. If we observed that there was one (see *Results*), we then compared Tests II and III to test if this winner–loser effect persisted (i.e. was still as strong 7 d later). We did not use Tests I and II to test for persistence because this comparison does not control for greater familiarity with the test apparatus in Test II.

### Encounter tests

Encounter tests were conducted in a 90-L glass aquarium (60 × 40 × 40 cm) with internal 3D-printed walls (Original Prusa i3 MK3S + 3D printer, Prusa Research, Praha, Czech Republic) used to form a funnel-shaped arena (Fig. 2). The arena contained a start area at one end which was connected by a corridor to a reward area at the opposite end. The focal male was introduced into a mesh cylinder in the start area with two “companion” juvenile mosquitofish in a transparent plastic cylinder alongside him (Fig. 2). Four large females with obvious gravid spots were housed in a 0.5 L transparent tank in the reward area. The presence of the “companion” juveniles meant that the focal male did not have to swim through the corridor to school with other fish. There was a transparent plastic cylinder at the center of the corridor that housed either a rival male, a juvenile, or was left empty. The rival male was significantly larger than the focal male (difference in SL: 1.61 mm ± 0.17 mm), which should reduce the propensity of a focal male to enter the corridor to reach the females. The empty cylinder was our control treatment. To test whether there was simply an effect of the presence of any conspecific, we used a juvenile in the cylinder as a second control. We denoted an “encounter area” 3 cm around the cylinder.

In each trial, following a 5-min acclimation period, the mesh cylinder was carefully raised so that the focal male could swim freely, and school with the companion juveniles, or enter the corridor and eventually reach the reward area. We recorded for each focal male: (1) initiation time: the time taken to enter the corridor; (2) encounter time: the time spent ≤ 3 cm (equivalent to 1 SL of the male) from the cylinder in the corridor; and (3) total trial time: the time taken from release until reaching the reward area (see Supplementary Material for details). The trial concluded when the male entered the reward area, or when 10 min had elapsed. Each male underwent 3 trials (one per encounter type) within a day, with a 5-min acclimation period between trials. The order of encounter types was randomized. Two size-matched fish (a winner and a loser) were tested on the same day, encountered the 3 cylinder types in the identical order, and were tested with the same 2 juveniles, 4 females, and a larger rival male. Focal males tested on the same day were presented with the same set of females, juveniles, and rivals. All behavior was recorded on a 5MP dome camera (CCTV Central, Victoria, Australia) mounted above the tank. The videos were analyzed blind to whether males were winners or losers.

### Statistical analysis

A 2-step analysis was conducted for each of the recorded behaviors. First, we examined the likelihood that the male: (1) left the start area, (2) entered the encounter area, and (3) entered the reward area. We ran separate generalized linear mixed models (GLMMs) with binomial error (*glmmTMB* package). Second, for those males who exhibited the above behaviors, we ran individual GLMMs (with Gaussian error) to analyze the time spent on each behavior. We log-transformed the time data to fulfill the model assumptions. Model residuals of all GLMMs were checked using the *DHARMa* package (Hartig 2020). Contest outcome (winner, loser), timing relative to contest/trial experience (Test I, Test II, Test III), and encounter type (male rival, juvenile, empty cylinder) were treated as fixed factors, and their 3-way and 2-way interactions were included in the initial model. Male identity was treated as a random factor to control for repeated measures from the same male. We also treated the size-matched winner/loser pair’s identity as a random factor to account for both males facing the same stimulus fish, on the same day, with the same encounter type order.

We disentangled the effect of test order (first or second test) and timing (time elapsed since the 24-h contest treatment) using contrast matrices derived from the summary results of the GLMMs. For each response variable, we first investigated whether test order altered the effect of contest outcome on the response to different encounter types (i.e. the 3-way interaction) by only considering data from Test I (first test immediately after contest experience) and Test III (second test immediately after contest experience). As there were no significant differences between Test I and III, we combined the data to test whether the immediate effect of contest outcome depended on the encounter type (i.e. a significant 2-way interaction). If the 2-way interaction was significant for a given response variable, we reported the effect of contest outcome for each encounter type (empty, rival, juvenile); otherwise, we reported the main effect of contest outcome. If there was a significant effect of contest outcome for a given response variable, we further compared data from Test II (second test, 1 wk after contest experience) to Test III (second test, immediately after contest experience) using the same approach to test for the persistence of the observed winner/loser effect. *P* values and effect estimates were calculated using generalized linear hypothesis tests in *multicomp* package (Hothorn et al. 2023).

Although it was not the focus of our study, we also tested the repeatability of boldness between Tests I and II. Sheltering behavior in a neutral environment is commonly used to measure boldness, so we ran repeatability tests on whether males left the start area for the non-agonistic/control treatments (i.e. an empty cylinder and a cylinder with a juvenile). We treated the response as binary (i.e. left/did not leave the start area) and male ID as a random factor in the repeatability tests (using the rptR package) (Stoffel et al. 2017).

All analyses were conducted in R version 4.2.1. Results are presented as mean ± s.e., with the significance level set at α = 0.05 (2 tailed).

## Results

### Winner–loser effect

There were no significant 3-way interactions in any of the analyses using data from Test I and Test III (all *P* > 0.10; Supplementary Table S1). We therefore pooled the data for all males that were tested immediately after their contest experience ended (i.e. from Tests I and III).

**Fig. 3.**
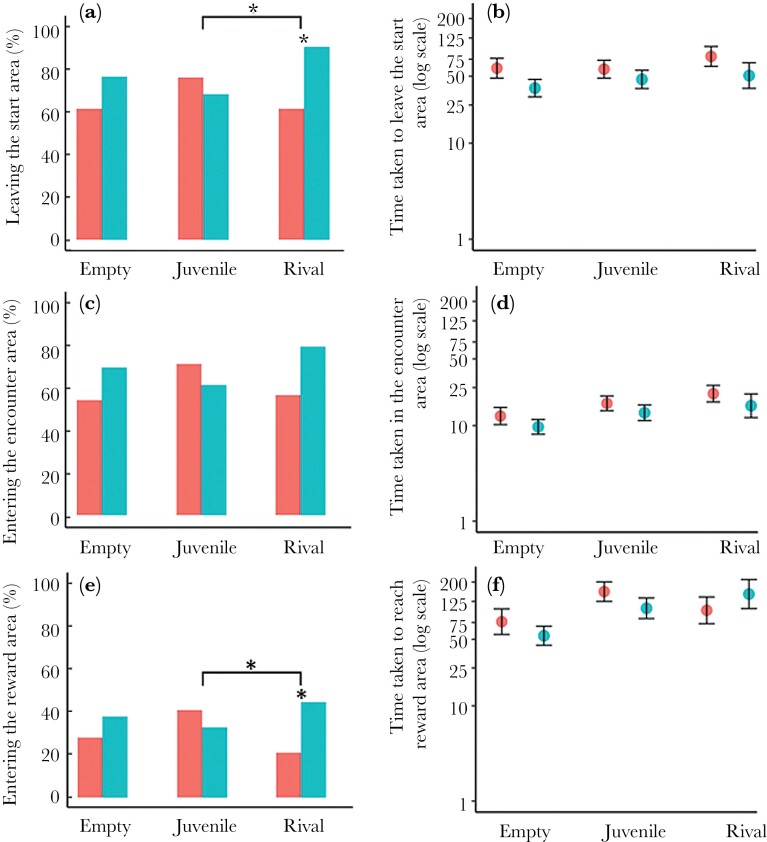
The difference between losers (red) and winners (blue) for males from Test I and Test III in the percentage of males: (a) leaving the start area, (c) entering the encounter area, (e) entering the reward area and the time (mean ± s.e.) taken on a logarithmic scale: (a) to leave the start area, (b) spent in the encounter area, and (c) to enter the reward area for the 3 encounter types (*n* = 46 pairs of winner/loser). Asterisks above encounter types indicate significant differences between winners and losers, while lines with asterisks indicate significant differences across encounter (*P < 0.05; non-significant differences are not indicated).

There was no effect of whether it was a male’s first or second test on the interaction between winning/losing and encounter type (Supplementary Table S1: non-significant 3-way interaction). Winners were significantly more likely than losers to leave the start area when facing a rival (*P* = 0.020) but not when facing a juvenile (*P* = 0.244) or an empty cylinder (*P* = 0.930) (Fig. 3a). Winners and losers differed significantly in the likelihood of leaving the start area when confronted with a rival rather than a juvenile (*P* = 0.024), but did not differ in their response to a rival and an empty cylinder (*P* = 0.173) (Fig. 3a). Of those males that left the start area, a winning/losing experience did not significantly affect the time taken to leave the start area, nor was there any interaction with encounter type or test order (Supplementary Table S1) (Fig. 3b).

Whether or not a male entered the encounter area, and the time then spent there if he did, was not significantly affected by winning/losing, nor by its interactions with test order or encounter type (Fig. 3c,d; Supplementary Table S1).

There was a marginally significant interaction between contest outcome and encounter type on the likelihood of reaching the reward area. Winners were more likely than losers to enter the reward area when a rival was present (*P* = 0.049), but not when facing a juvenile (*P* = 0.819), or an empty container (*P* = 0.609) (Fig. 3e). There was a significant effect of contest outcome on whether a male entered the reward area in the presence of a rival versus a juvenile (*P* = 0.045), but no significant difference when facing a rival or an empty cylinder (*P* = 0.595) (Fig. 3e). Finally, there was no significant effect of contest outcome, or any interactive effects, on the time taken to reach the reward area (Supplementary Table S1) (Fig. 3f).

Our binary measure of boldness was not significantly repeatable between Test I and Test II in either the empty cylinder (repeatability = 0.04, *P* = 0.375) or the juvenile treatment (repeatability = 0, *P* = 1).

### Persistence of winner–loser effect

**Fig. 4.**
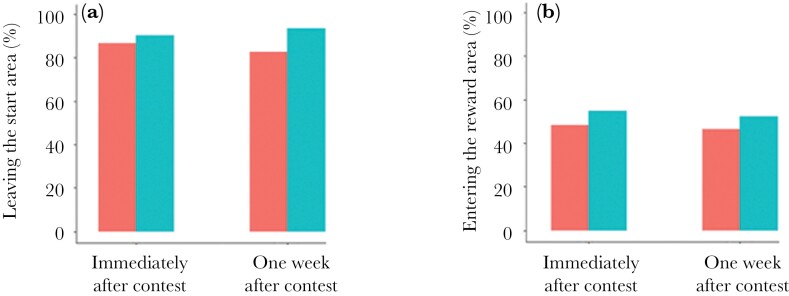
The percentage of males: (a) leaving the start area and (b) entering the reward area in second encounter tests either immediately after contest experience (Test III) or 1 wk after contest experience (Test II). Losers = red; winners = blue.

As winners and losers differed in the likelihood of leaving the start area and entering the reward area, we assessed the persistence of these two winner*–*loser effects by comparing Tests II and III. There was no evidence of a difference in whether or not a male left the start area or entered the reward area between the 2 tests (all *P* > 0.30; Supplementary Table S4), indicating that the winner/loser effect did not detectably decline over a week (Fig. 4).

## Discussion

We tested if there was a winner*–*loser effect for male *G. holbrooki* based on their response to potential rivals before they could approach females. We measured the propensity and time taken: to leave a “safe” start area, to interact with a rival male (or control), and to reach the females. Winners were significantly more likely than losers to leave the start area when a larger rival male was between them and the females, but not where there was a juvenile or no fish between them and the females. Likewise, winners were significantly more likely than losers to reach the females when a larger rival male was between them and the females, but not where there was a juvenile or no fish between them and the females. These two winner*–*loser effects were still present when males were tested again 1 wk later.

### Winner–loser effect in mosquitofish

The tendency to leave a shelter in a neutral situation (i.e. in our study when facing the empty cylinder or a juvenile) is often used as a measure of ‘boldness’ (Magnhagen and Borcherding 2008; Riesch et al. 2009; Leite et al. 2022). Conversely, in agonistic contests, the tendency to engage in aggressive interactions is defined as “aggressiveness” (Logue et al. 2011). Therefore, when exposed to a larger rival in the cylinder, we assume that leaving the shelter and approaching potential mates reflect risk-taking behavior during aggressive interactions. In our study, we found that male *G. holbrooki* that won fights appeared to be more aggressive than losers (i.e. more likely to leave the start zone when faced with a larger rival). However, there was no evidence of a winner*–*loser effect on boldness.

Positive natural correlations between boldness and aggressiveness have been documented in many species (e.g. 3-spined sticklebacks *Gasterosteus aculeatus*, Aubin-Horth et al. 2012; house wrens *Troglodytes aedon*, Barnett et al. 2012). However, the plasticity of these two behavioral traits when facing opponents can vary significantly. For instance, in male crayfish (*Faxonius virilis*), losing experiences can reduce their boldness (Mathews 2021), whereas in mangrove killifish (*Kryptolebias marmoratus*), winners become significantly more aggressive, but not bolder, after contests (Chen et al. 2022). In some asymmetric contests, the winner*–*loser effects on boldness and aggressiveness differ between roles (i.e. attackers and defenders) (Courtene-Jones and Briffa 2014). Although both boldness and aggressiveness are often correlated, many species, including eastern mosquitofish, exhibit varying plasticity in these traits after contests (Axling et al. 2023). As such, researchers should be cautious about inferring the relationship among boldness, aggressiveness, and the likelihood of winning fights in future studies.

Aggressiveness plays a crucial role in maintaining dominance and access to resources vital for fitness. For instance, aggressive behavior is linked to many fitness-related goals, including acquiring food resources, defending offspring, and securing mating opportunities (Gottreich et al. 2001). In long-lived carnivorous birds, nestling aggression is common as they compete with their broodmates for food (Redondo et al. 2019). Ural owls (*Strix uralensis*) increase their aggressiveness levels in response to increased prey abundance (Kontiainen et al. 2009). Additionally, some species exhibit greater aggression during reproduction, such as the Adriatic dwarf goby (*Knipowitschia panizzae*) (Nonnis Marzano and Gandolfi 2001), and the Ural owl (Kontiainen et al. 2009). Previous studies have also demonstrated that higher levels of aggressiveness enhance mating success by increasing males’ competitiveness for reproduction-related resources and, consequently, their attractiveness to females (Potapov et al. 2010; Hubená et al. 2021). For instance, in the water striders (*Aquarius remiges*), the water voles (*Arvicola terrestris*), and the house mouses (*Mus musculus*), more aggressive males often have higher mating success (Potapov et al. 2010; Sih et al. 2014). Similarly, winners in our study were more likely than losers to bypass a large rival in the cylinder and approach females. While the transparent cylinder in the corridor could potentially obscure visual access to females, it seems unlikely to be the case. A recent study on mosquitofish conducted in similar arenas (Vinogradov et al. 2022) suggests that *G. holbrooki* can see other fish through transparent barriers placed between the focal individuals and conspecific rewards. Male mosquitofish have been reported to exhibit a winner*–*loser effect when they freely compete for mates, whereby winners are more successful (Harrison et al. 2018, 2023). Our findings further suggest that a winner*–*loser effect on mating success could exist even before males directly fight because of a greater willingness of winners to approach females.

A winner*–*loser effect on general aggressiveness has been reported in several species (Logue et al. 2011), but few studies have examined individual male’s aggressive intent at the early stage of contests. Most fish studies use displays at short distances and physical contacts, such as attacks and nipping, as indicators of aggressiveness (e.g. mangrove rivulus *Kryptolebias marmoratus*, Chang et al. 2012; zebrafish *Danio rerio*, Ariyomo and Watt 2012; Amazon molly *Poecilia formosa*, Doran et al. 2019). It is worth noting that the majority of contests are resolved without physical fighting as the cost of attack behavior can be extremely high (Luo et al. 2017). Therefore, exploring an individual’s decision-making and early stages of aggressiveness is often crucial to measure their aggressive intent accurately. In our study, we measured the time males took to leave a shelter and approach a larger rival before any fighting occurs, which provided valuable insights into a male’s willingness to be aggressive. Additionally, the larger rival was kept in a transparent cylinder, ensuring that the behavior of the focal fish was not directly influenced by their rival’s fighting behavior. Further studies investigating how winning or losing past fights affects other sexually selected fitness components (e.g. ability to locate mates, ejaculate competitiveness) would be valuable.

Intriguingly, no benefit of winning past encounters was detected when a rival was absent in the trials. Only a handful of studies have investigated the winner*–*loser effect in the absence of a rival. For instance, in rainbow trout (*Oncorhynchus mykiss*), both winning and losing experiences led to a shorter latency to investigate a novel object when no other individuals were present (Frost et al. 2007). In fruit flies (*Drosophila melanogaster*) (Teseo et al. 2016) and crickets (*Velarifictorus asperses*) (Zeng et al. 2018), males that lost a fight reduced their courtship effort when alone with a female. Our current study suggests that male mosquitofish seem to use information from previous fights to adjust their subsequent behaviors, but this adjustment is contingent upon the social environment.

### Persistence of winner–loser effect

Winning past fights significantly increased a male’s tendency both to leave the start area and to reach prospective mates when tested immediately after he had spent 24 h interacting with a rival. We further demonstrated that these winner*–*loser effects did not decline significantly after a week. Our study contributes novel insights to the limited research on persistent winner*–*loser effects, building upon findings from Momohara et al. (2016) on crayfish (*Procambarus clarkia*), where a winner*–*loser effect lasted for 10 d, and from Lan and Hsu (2011) on mangrove killifish, where winner*–*loser dynamics persisted for over a month. Notably, many species exhibit short-term winner*–*loser effects lasting less than a day, as seen in pumpkinseed sunfish (*Lepomis gibbosus*) (Chase et al. 1994) and rusty crayfish (*Orconectes rusticus*) (Bergman et al. 2003). Our results add to the few available studies that show a persistent winner*–*loser effect.

The prolonged effect observed in mosquitofish was unexpected given their natural tendency to school together in shallow freshwater environments, leading to a higher rate of social encounters (Ward 2012). We would expect species with a short breeding season and/or a short lifespan (i.e. mosquitofish) to prioritize recent information over past experiences, resulting in only short-term effects of contest outcomes (Hsu et al. 2006; Kasumovic et al. 2010). In our study, males were held in isolation between tests, so the relevance of the observed longer-term winner*–*loser effect for males in the wild is unclear. Further studies that test for this effect in the presence of frequent social encounters are necessary. Nonetheless, our findings suggest that males do possess mechanisms to store information about their fighting ability for at least a week.

Long-term persistence of winner*–*loser effects might arise from conflict-induced physiological changes. Male vertebrates often respond to social challenges by increasing their plasma androgen concentrations to boost their aggression and prospect of winning a fight (Moore et al. 2020). Elevated androgen levels can sustain aggressive behavior even after the initial challenge has ended (Deviche et al., 2012). Other hormones may also contribute to a longer-term winner*–*loser effects. For instance, in crayfish, where a winner effect lasted for over 10 d, serotonin seemed to mediate the winner effect, while octopamine might trigger the loser effect (Momohara et al. 2013, 2016). In rainbow trout, losers consistently had higher levels of cortisol expression after contests, whereas cortisol concentrations decreased rapidly in winners (Øverli et al. 2000). Although changes in hormone levels are generally considered the basis of longer-term behavioral adjustment (Moore et al. 2020), the role of endocrine mediators in winner*–*loser effects is often unknown. Our findings suggest that *G. holbrooki* could be a model species for such studies (see also Harrison et al. 2018, 2023).

## Conclusion

Male *G. holbrooki* that had previously won fights exhibited a greater likelihood of leaving a shelter and approaching females in the presence of a larger rival. Importantly, this winner*–*loser effect depended on the social environment and was absent in a non-competitive scenario, which suggests that males actively adjust their aggressiveness based on past fighting experience. Notably, this effect appeared at the very early stage of male*–*male contests and persisted for at least 7 d. Our study highlights the potential for winning/losing experiences to have effects that extend beyond future fights and to affect other fitness components, such as mating.

## Supplementary Material

arae037_suppl_Supplementary_Tables_S1-S4

## Data Availability

Analyses reported in this article can be reproduced using the data provided by Zang et al. (2024), which had been submitted in Dryad (https://datadryad.org/stash/dataset/doi:10.5061/dryad.s4mw6m9dv).
