# Supplementary material for: Does losing reduce the tendency to engage with rivals to reach mates? An experimental test
Source: Behav Ecol. 2024 May 3;35(4):arae037. doi: 10.1093/beheco/arae037 (PMC11107846; doi:10.1093/beheco/arae037)
Supplement: arae037_suppl_Supplementary_Tables_S1-S4 [file arae037_suppl_supplementary_tables_s1-s4.docx]

**Does losing reduce the tendency to engage with rivals to reach mates? An experimental test**

**Supplementary material**

*Measurement of response variables*

We recorded (i) time taken to leave the start area: the time males spent until entering the corridor, marked by the snout tip crossing the boundary of the start area; (ii) time spent in the encounter time: the time males spent within 3 cm (equivalent to 1 SL of the male) of the cylinder in the corridor, starting from when the snout tip entered the area until the body fully left the area; and (iii) total trial time: the time males spent until entering the reward area. The trial concluded either when the male entered the reward area or when 10 minutes elapsed.

Table S1. Full output of the general linear hypotheses tests to analyse the winner-loser effect (Tests I and III) on initiation. In this analysis, (a) whether males left the start area was examined for the effects of various factors with binomial error distribution and (b) time taken to leave the start was tested with Gaussian distribution. All analysis contains three-way interaction (contest outcome* encounter type* test), two-way interactions (Contest outcome* encounter type and Contest outcome* test) and the effect of contest outcome. The Log odds ratio for binomial distribution, estimate for Gaussian distribution, standard error, z-value and p-value for each general linear hypothesis are included in this table. The significance level is indicated in bold font for p-values less than 0.05.

| **(a) Whether males left the start area (binomial)** | | | | | |
| --- | --- | --- | --- | --- | --- |
| Three-way interaction (Contest outcome* encounter type*test) | (Difference between two-way interaction in Test III and in Test I) | | | | |
|  |  | Difference in log odds ratio difference | Std. Error | z-value | p-value |
|  | (juvenile/empty) | 0.375 | 1.979 | 0.190 | 1.000 |
|  | (rival/empty) | 1.988 | 2.086 | 0.953 | 0.945 |
|  | (rival/juvenile) | -1.612 | 2.040 | -0.790 | 0.978 |
| Two-way interaction (Contest outcome* encounter type) | (averaging the two-way interactions in Test I and Test III) | | | | |
|  |  | Difference in log odds ratio | Std. Error | z-value | p-value |
|  | (juvenile/empty) | -1.786 | 0.998 | -1.789 | 0.173 |
|  | (rival/empty) | 0.924 | 1.040 | 0.888 | 0.648 |
|  | (rival/juvenile) | -2.710 | 1.032 | -2.627 | **0.024** |
| Contest outcome | (Winner/losers in different encounter types) | | | | |
|  |  | Log odds ratio | Std. Error | z-value | p-value |
|  | (empty) | 1.3744 | 0.7980 | 1.722 | 0.2442 |
|  | (juvenile) | -0.4119 | 0.7600 | -0.542 | 0.9301 |
|  | (rival) | 2.2985 | 0.8356 | 2.751 | **0.0204** |
|  | (overall) | 1.0870 | 0.5368 | 2.025 | 0.1324 |
| **(b) Time taken to leave the start area (Gaussian)** | | | | | |
| Three-way interaction (Contest outcome* encounter type*test) | (Difference between two-way interaction in Test III and in Test I) | | | | |
|  |  | Difference of 2-way interaction effect | Std. Error | z-value | p-value |
|  | (juvenile/empty) | -0.072 | 0.731 | -0.098 | 1.000 |
|  | (rival/empty) | 0.201 | 0.730 | 0.275 | 1.000 |
|  | (rival/juvenile) | -0.273 | 0.718 | -0.380 | 1.000 |
| Two-way interaction (Contest outcome* encounter type) | (averaging the two-way interactions in Test I and Test III) | | | | |
|  |  | Estimate of interaction | Std. Error | z-value | p-value |
|  | (juvenile/empty) | 0.231 | 0.366 | 0.632 | 0.803 |
|  | (rival/empty) | 0.187 | 0.365 | 0.514 | 0.865 |
|  | (rival/juvenile) | 0.044 | 0.359 | 0.122 | 0.992 |
| Contest outcome | (Winner/losers in different encounter types) | | | | |
|  |  | Estimate | Std. Error | z-value | p-value |
|  | (empty) | 1.052 | 0.630 | 1.670 | 0.273 |
|  | (juvenile) | -0.402 | 0.616 | -0.652 | 0.888 |
|  | (rival) | 1.460 | 0.628 | 2.324 | 0.067 |
|  | (overall) | 0.703 | 0.406 | 1.731 | 0.244 |

Table S2. Full output of the general linear hypotheses tests to analyse the winner-loser effect (Tests I and III) on encounter. In this analysis, (a) whether males entered the encounter area was examined for the effects of various factors with binomial error distribution and (b) time spent in the encounter area was tested with Gaussian distribution. All analysis contains three-way interaction (contest outcome* encounter type* test), two-way interactions (Contest outcome* encounter type and Contest outcome* test) and the effect of contest outcome. The Log odds ratio for binomial distribution, estimate for Gaussian distribution, standard error, z-value and p-value for each general linear hypothesis are included in this table. The significance level is indicated in bold font for p-values less than 0.05.

| **(a) Whether males entered the encounter area (binomial)** | | | | | |
| --- | --- | --- | --- | --- | --- |
| Three-way interaction (Contest outcome* encounter type*test) | (Difference between two-way interaction in Test III and in Test I) | | | | |
|  |  | Difference in log odds ratio difference | Std. Error | z-value | p-value |
|  | (juvenile/empty) | 0.254 | 1.636 | 0.155 | 1.000 |
|  | (rival/empty) | -0.203 | 1.646 | -0.123 | 1.000 |
|  | (rival/juvenile) | -0.457 | 1.634 | -0.280 | 1.000 |
| Two-way interaction (Contest outcome* encounter type) | (averaging the two-way interactions in Test I and Test III) | | | | |
|  |  | Difference in log odds ratio | Std. Error | z-value | p-value |
|  | (juvenile/empty) | -1.454 | 0.822 | -1.768 | 0.180 |
|  | (rival/empty) | 0.408 | 0.823 | 0.496 | 0.873 |
|  | (rival/juvenile) | 1.862 | 0.822 | 2.265 | 0.061 |
| Contest outcome | (Winner/losers in different encounter types) | | | | |
|  |  | Log odds ratio | Std. Error | z-value | p-value |
|  | (empty) | 1.052 | 0.630 | 1.670 | 0.273 |
|  | (juvenile) | -0.402 | 0.616 | -0.652 | 0.888 |
|  | (rival) | 1.460 | 0.628 | 2.324 | 0.067 |
|  | (overall) | 0.703 | 0.406 | 1.731 | 0.244 |
| **(b) Time spent in the encounter area (Gaussian)** | | | | | |
| Three-way interaction (Contest outcome* encounter type*test) | (Difference between two-way interaction in Test III and in Test I) | | | | |
|  |  | Difference of 2-way interaction effect | Std. Error | z-value | p-value |
|  | (juvenile/empty) | 1.058 | 0.709 | 1.493 | 0.538 |
|  | (rival/empty) | 0.341 | 0.709 | 0.481 | 0.988 |
|  | (rival/juvenile) | -0.717 | 0.687 | -1.044 | 0.818 |
| Two-way interaction (Contest outcome* encounter type) | (averaging the two-way interactions in Test I and Test III) | | | | |
|  |  | Estimate of interaction | Std. Error | z-value | p-value |
|  | (juvenile/empty) | 0.034 | 0.354 | 0.096 | 0.995 |
|  | (rival/empty) | 0.171 | 0.354 | 0.484 | 0.879 |
|  | (rival/juvenile) | 0.137 | 0.343 | 0.400 | 0.916 |
| Contest outcome | (Winner/losers in different encounter types) | | | | |
|  |  | Estimate | Std. Error | z-value | p-value |
|  | (empty) | -0.260 | 0.274 | -0.949 | 0.725 |
|  | (juvenile) | -0.226 | 0.259 | -0.873 | 0.772 |
|  | (rival) | -0.089 | 0.258 | -0.346 | 0.981 |
|  | (overall) | -0.260 | 0.274 | -0.949 | 0.725 |

Table S3. Full output of the general linear hypotheses tests to analyse the winner-loser effect (Tests I and III) on total trial solving time. In this analysis, (a) whether males entered the reward area was examined for the effects of various factors with binomial error distribution and (b) time taken to reach the reward area was tested with Gaussian distribution. All analysis contains three-way interaction (contest outcome* encounter type* test), two-way interactions (Contest outcome* encounter type and Contest outcome* test) and the effect of contest outcome. The Log odds ratio for binomial distribution, estimate for Gaussian distribution, standard error, z-value and p-value for each general linear hypothesis are included in this table. The significance level is indicated in bold font for p-values less than 0.05.

| **(a) Whether males entered the reward area (binomial)** | | | | | |
| --- | --- | --- | --- | --- | --- |
| Three-way interaction (Contest outcome* encounter type*test) | (Difference between two-way interaction in Test III and in Test I) | | | | |
|  |  | Difference in log odds ratio difference | Std. Error | z-value | p-value |
|  | (juvenile/empty) | 0.419 | 1.432 | 0.293 | 0.998 |
|  | (rival/empty) | 1.160 | 1.487 | 0.780 | 0.929 |
|  | (rival/juvenile) | 0.740 | 1.440 | 0.514 | 0.984 |
| Two-way interaction (Contest outcome* encounter type) | (averaging the two-way interactions in Test I and Test III) | | | | |
|  |  | Difference in log odds ratio | Std. Error | z-value | p-value |
|  | (juvenile/empty) | -1.007 | 0.718 | -1.403 | 0.339 |
|  | (rival/empty) | 0.723 | 0.744 | 0.971 | 0.595 |
|  | (rival/juvenile) | 1.729 | 0.725 | 2.385 | **0.045** |
| Contest outcome | (Winner/losers in different encounter types) | | | | |
|  |  | Log odds ratio | Std. Error | z-value | p-value |
|  | (empty) | 0.607 | 0.535 | 1.135 | 0.609 |
|  | (juvenile) | -0.400 | 0.499 | -0.801 | 0.819 |
|  | (rival) | 1.330 | 0.542 | 2.452 | **0.049** |
|  | (overall) | 0.512 | 0.315 | 1.626 | 0.303 |
| **(b) Time taken to reach the reward area (Gaussian)** | | | | | |
| Three-way interaction (Contest outcome* encounter type*test) | (Difference between two-way interaction in Test III and in Test I) | | | | |
|  |  | Difference of 2-way interaction effect | Std. Error | z-value | p-value |
|  | (juvenile/empty) | 2.378 | 0.918 | 2.590 | 0.063 |
|  | (rival/empty) | 0.073 | 1.005 | 0.073 | 1.000 |
|  | (rival/juvenile) | -2.305 | 0.944 | -2.441 | 0.092 |
| Two-way interaction (Contest outcome* encounter type) | (averaging the two-way interactions in Test I and Test III) | | | | |
|  |  | Estimate of interaction | Std. Error | z-value | p-value |
|  | (juvenile/empty) | -0.063 | 0.465 | -0.135 | 0.990 |
|  | (rival/empty) | 0.770 | 0.499 | 1.544 | 0.270 |
|  | (rival/juvenile) | 0.833 | 0.466 | 1.788 | 0.173 |
| Contest outcome | (Winner/losers in different encounter types) | | | | |
|  |  | Estimate | Std. Error | z-value | p-value |
|  | (empty) | -0.340 | 0.385 | -0.881 | 0.761 |
|  | (juvenile) | -0.402 | 0.343 | -1.172 | 0.568 |
|  | (rival) | 0.431 | 0.390 | 1.105 | 0.614 |
|  | (overall) | -0.104 | 0.252 | -0.411 | 0.967 |

Table S4. Full output of the general linear hypotheses tests to analyse the persistence of winner-loser effects (Tests II and III) on (a) whether males left the start area and (b) whether males reached the reward area with binomial error. All analysis contains three-way interaction (contest outcome* encounter type* test), two-way interactions (Contest outcome* encounter type and Contest outcome* test) and the effect of contest outcome. The Log odds ratio for binomial distribution, standard error, z-value and p-value for each general linear hypothesis are included in this table. The significance level is indicated in bold font for p-values less than 0.05.

| **(a) Whether males left the start area (binomial)** | | | | | |
| --- | --- | --- | --- | --- | --- |
| Three-way interaction (Contest outcome* encounter type*test) | (Difference between two-way interaction in Test III and in Test I) | | | | |
|  |  | Difference in log odds ratio difference | Std. Error | z-value | p-value |
|  | (juvenile/empty) | -2.844 | 2.211 | -1.286 | 0.675 |
|  | (rival/empty) | -0.267 | 2.301 | -0.116 | 1.000 |
|  | (rival/juvenile) | 2.576 | 2.278 | 1.131 | 0.770 |
| Two-way interaction (Contest outcome* encounter type) | (averaging the two-way interactions in Test I and Test III) | | | | |
|  |  | Difference in log odds ratio | Std. Error | z-value | p-value |
|  | (juvenile/empty) | -0.552 | 1.103 | -0.501 | 0.936 |
|  | (rival/empty) | 0.064 | 1.149 | 0.055 | 1.000 |
|  | (rival/juvenile) | 1.961 | 2.233 | 0.878 | 0.740 |
| Contest outcome | (Winner/losers in different encounter types) | | | | |
|  |  | Log odds ratio | Std. Error | z-value | p-value |
|  | (empty) | 1.004 | 0.864 | 1.162 | 0.577 |
|  | (juvenile) | 0.452 | 0.845 | 0.535 | 0.939 |
|  | (rival) | -0.262 | 1.1146 | -0.235 | 0.996 |
|  | (overall) | 0.841 | 0.5800 | 1.450 | 0.388 |
| **(b) whether males reached the reward area (binomial)** | | | | | |
| Three-way interaction (Contest outcome* encounter type*test) | (Difference between two-way interaction in Test III and in Test I) | | | | |
|  |  | Difference in log odds ratio difference | Std. Error | z-value | p-value |
|  | (juvenile/empty) | -1.417 | 1.309 | -1.083 | 0.798 |
|  | (rival/empty) | 0.766 | 1.333 | 0.575 | 0.976 |
|  | (rival/juvenile) | 2.184 | 1.340 | 1.630 | 0.450 |
| Two-way interaction (Contest outcome* encounter type) | (averaging the two-way interactions in Test I and Test III) | | | | |
|  |  | Difference in log odds ratio | Std. Error | z-value | p-value |
|  | (juvenile/empty) | -0.089 | 0.653 | -0.136 | 0.999 |
|  | (rival/empty) | 0.919 | 0.668 | 1.376 | 0.407 |
|  | (rival/juvenile) | 1.034 | 1.547 | 0.668 | 0.865 |
| Contest outcome | (Winner/losers in different encounter types) | | | | |
|  |  | Log odds ratio | Std. Error | z-value | p-value |
|  | (empty) | -0.0233 | 0.4731 | -0.049 | 1.000 |
|  | (juvenile) | -0.1118 | 0.4728 | -0.237 | 0.997 |
|  | (rival) | 0.1941 | 0.6997 | 0.277 | 0.995 |
|  | (overall) | 0.2536 | 0.2889 | 0.878 | 0.784 |
